# DNA methylation is stable during replication and cell cycle arrest

**DOI:** 10.1038/srep17911

**Published:** 2015-12-09

**Authors:** Amy R. Vandiver, Adrian Idrizi, Lindsay Rizzardi, Andrew P. Feinberg, Kasper D. Hansen

**Affiliations:** 1 Center for Epigenetics, Johns Hopkins University School of Medicine; 2 Department of Medicine, Johns Hopkins University School of Medicine; 3 McKusick-Nathans Institute of Genetic Medicine, Johns Hopkins University School of Medicine; 4 Department of Biostatistics, Johns Hopkins Bloomberg School of Public Health.

## Abstract

DNA methylation is an epigenetic modification with important functions in development. Large-scale loss of DNA methylation is a hallmark of cancer. Recent work has identified large genomic blocks of hypomethylation associated with cancer, EBV transformation and replicative senescence, all of which change the proportion of actively proliferating cells within the population measured. We asked if replication or cell-cycle arrest affects the global levels of methylation or leads to hypomethylated blocks as observed in other settings. We used fluorescence activated cell sorting to isolate primary dermal fibroblasts in G0, G1 and G2 based on DNA content and Ki67 staining. We additionally examined G0 cells arrested by contact inhibition for one week to determine the effects of extended arrest. We analyzed genome wide DNA methylation from sorted cells using whole genome bisulfite sequencing. This analysis demonstrated no global changes or large-scale hypomethylated blocks in any of the examined cell cycle phases, indicating that global levels of methylation are stable with replication and arrest.

DNA methylation is an epigenetic modification that has important functions in mammalian development. It consists of addition of a 5’ methyl group to the cytosine base. This modification is most frequently found in the context of CpG dinucleotides, where it can be placed by three methyltransferase enzymes: DNMT1, the maintenance methyltransferase that re-establishes the methylation pattern following DNA replication, and DNMT3a and DNMT3b, which function in *de novo* methylation^1^. The presence of DNA methylation in gene promoters and enhancers decreases gene expression, likely through alterations of local DNA structure and prevention of transcription factor binding^2^. Changes in methylation are associated with aging, oncogenesis and other diseases^3^,^4^,^5^,^6^,^7^,^8^.

Several molecular processes, including gene expression and chromatin structure, are known to change through the cell cycle^9^,^10^,^11^. These changes result in unwanted variation between measures on bulk, unsynchronized cells. Computational approaches have been suggested to control for this variation^12^,^13^.

The extent to which DNA methylation changes throughout the cell cycle is currently unknown. Previous studies of methylation during the cell cycle have focused on the maintenance of methylation during DNA replication. The maintenance methyltransferase, DNMT1, localizes to newly synthesized DNA and is associated with the replication complex during S-phase, and with various other transcription factors during G0/G1 and G2/M phases^14^. A previous study in HeLa cells reported increased methylation during S phase using immunofluorescence and HPLC^15^. By contrast, a more recent study using flow cytometry to quantify 5-methylcytosine (5 mC) in two cancer cell lines found no differences in the ratio of 5 mC staining to DNA content between G1 and S phase, but noted a lag in early G2/M before the maximum levels of 5 mC were observed^16^. While these studies provide valuable insights into the methylation of newly synthesized DNA, both studies focus on changes during DNA synthesis, and neither provides region specific data.

We have previously reported the presence of large hypomethylated domains, encompassing up to two thirds of the genome, termed “hypomethylated blocks”, in colon cancer samples and associated with EBV transformation of lymphocytes, indicating large-scale epigenetic structural changes^17^,^18^,^19^. These blocks of hypomethylation occur in gene-poor regions and overlap strongly with heterochromatin, lamina-associated domains (LADs) and so-called partial methylated domains (PMDs) – regions of intermediate methylation in IMR90 cells^20^. A single cell can be methylated (100% methylation), hemimethylated (50% methylation), methylated in an allele specific manner (50% methylation) or unmethylated (0% methylation) at a given location. Thus, the consistent observation of regions with intermediate methylation levels suggests that methylation in these areas is highly variable within cell populations. Intriguingly, large regions of hypomethylation are also observed in cells approaching replicative senescence^21^. In this context, hypomethylation was found in regions that replicate late in S phase, concurrent with decreased expression and decreased localization of DNMT1 to these domains in late passage cells.

We note that in both the cancer and EBV studies, hypomethylated blocks are seen in conditions where there is also an increase in the proportion of cells that are actively proliferating. Colon cancer is associated with increased cell proliferation outside of the normal proliferative zone of colonic crypts as measured by Ki67 staining^22^,^23^. EBV transformation directly promotes proliferation of previously resting cells by inducing expression of early G1 regulators^24^. Normal tissues are often distinguished by differences in the percent of proliferating cells, so any differences in methylation attributed to proliferation may be relevant to studies of tissue specific methylation as well^25^. Given these observations, we asked whether the observed hypomethylated blocks might be attributed to changes in the proportion of actively dividing cells within the populations studied^14^,^15^,^16^.

In this study, we sought to more fully elucidate the genome-scale changes in DNA methylation associated with cell proliferation. We used early passage primary dermal fibroblasts to avoid any artifacts from long-term cell culture and isolated quiescent and proliferating cells using flow cytometry. We further subdivided the proliferating cells into G1 and G2 phases to identify methylation changes as a result of DNA replication. We observed strikingly high degree of correlation across cell cycle phases both within and between. We found no hypomethylated blocks or global changes in DNA methylation associated with proliferation.

## Results

**Figure 1:**
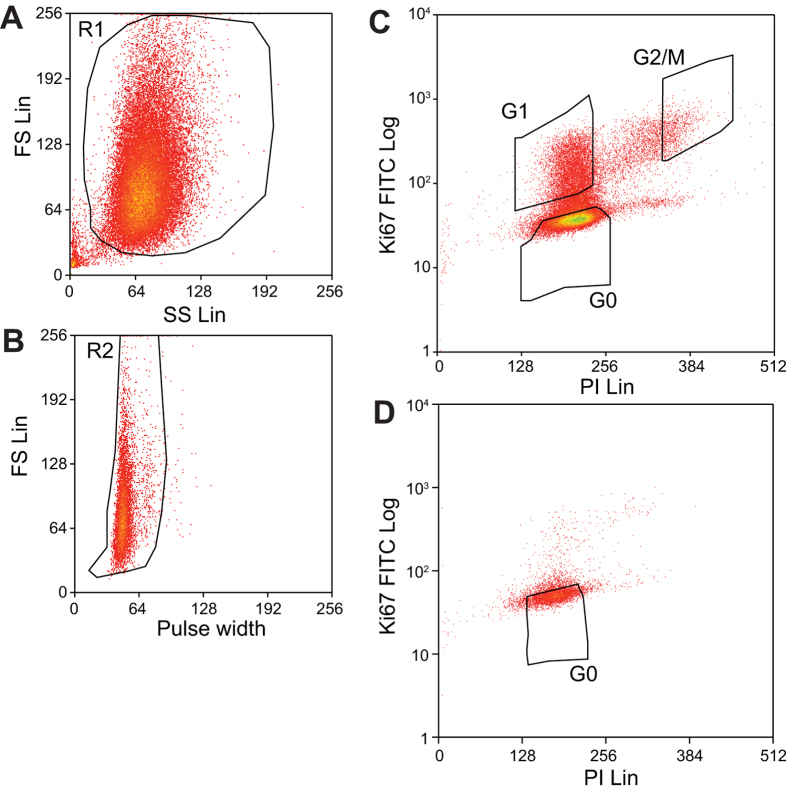
Sorting of fibroblasts based on Ki-67 expression and DNA content. (**A**) Live cells were selected using forward scatter and side scatter. (**B**) Single cells were selected using pulse width. (**C**) Cells from actively proliferating culture were sorted into “G0”: 2N DNA and Ki67 negative, “G1”: 2N DNA and Ki67 positive, “G2/M”:4N DNA and Ki67 positive. (**D**) Cells from culture arrested by contact inhibition for one week were predominantly “G0”.

We sought to identify potential genome scale changes in DNA methylation associated with cell proliferation. Working with 3 sets of early passage (P4) primary human dermal fibroblasts (details in Table 1), we used fluorescence activated cell sorting to separate cells into three groups based on staining with anti-Ki67 and Propidium Iodide: Live cells were gated using forward scatter and side scatter, single cells were gated using pulse width (Fig. 1A,B). Quiescent (G0) cells were sorted based on negative Ki67 staining and 2N DNA and represented 46-71% of live cells; G1 cells were identified as Ki67 positive and 2N DNA and represented 5-21% of live cells; G2/M cells were identified as Ki67 positive and 4N DNA and represented 3-5% of live cells (Fig. 1C). In addition, each set of primary cells was arrested by contact inhibition for 1 week to examine the influence of extended quiescence. After one week, 0.05-0.6% of all cells were in G2/M. Ki67 negative, 2N DNA cells were again isolated (Fig. 1D).

To gain unbiased genome wide information about DNA methylation in these samples, we performed whole genome bisulfite sequencing (WGBS). We generated sequencing data to a depth of 5.3–8.6× per sample and analyzed it using the BSmooth algorithm, which was designed for analyzing low-coverage WGBS data and has been demonstrated to accurately estimate methylation levels at single-base pair resolution by borrowing information from nearby CpGs^26^. After filtering reads with low quality measures, we obtained measurements for an average of 25,425,530 CpGs per sample. We focused our analysis on a common set of 23,527,039 CpGs (83% of the methylome) assayed sufficiently well across our experiment (see Methods). Bisulfite conversion was assessed using spiked in lambda phage and ranged from 99.67–99.71% (Details in Methods, Table 2).

**Figure 2:**
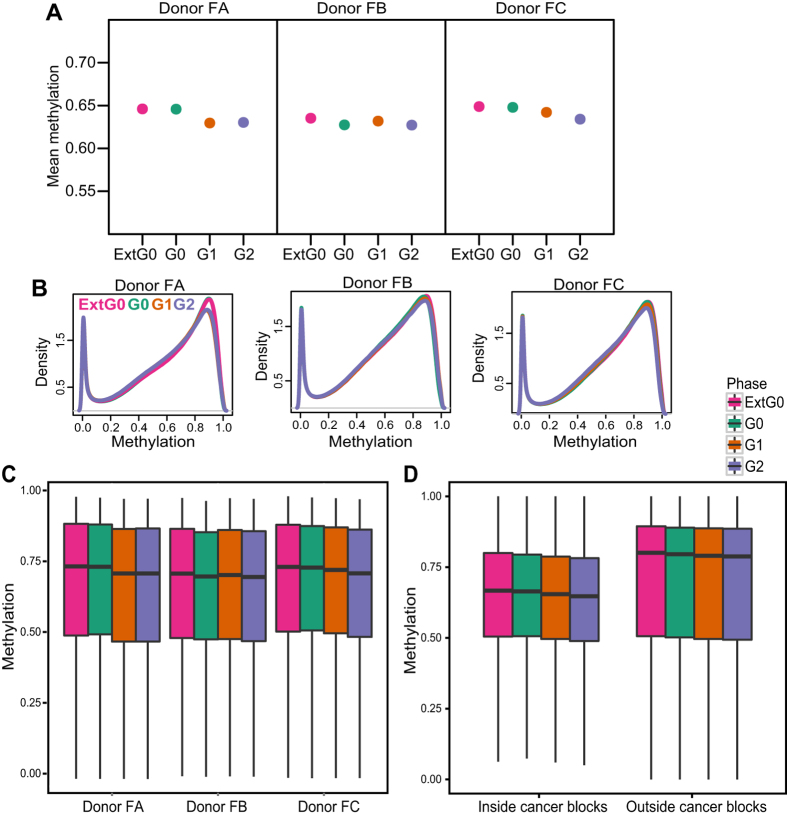
Global methylation is consistent after replication and cell cycle arrest. (**A**) Mean methylation for each cell cycle phase from each donor profiled. (**B**) Distribution of high-frequency smoothed methylation values from CpGs with sufficient coverage from whole genome bisulfite sequencing (WGBS) for each donor profiled. (**C)** Distribution of high-frequency smoothed methylation values from all CpGs analyzed for each donor and cell cycle phase analyzed. Outliers have been removed for plotting. **(D)** Distribution of mean high-frequency smoothed methylation values across all donors from CpGs within and outside the regions previously identified as hypomethylated blocks in colon cancer for each phase profiled. Outliers have been removed for plotting.

First, we examined the genome-wide distribution of DNA methylation in each cell cycle phase, specifically extended (1 week) G0, G0, G1 and G2. Figure 2A shows average methylation across the genome, for each cell cycle phase and donor. Analysis of variance indicates that variation of mean methylation between the cell cycle phases is not larger than variation within the phases (p = 0.243). However, we noticed a tendency to a decrease in average methylation across cell cycle phases, most apparent in donor FC. A paired t-test comparing the means of the most visibly different groups, extended G0 and G2, shows only marginal significance (p = 0.034). The overall methylation change is less than 1%. Figure 2B show the genomewide distribution of methylation for samples from each donor with the characteristic bimodal shape with peaks close to 0% and 100% methylation; there is no change in shape associated with cell cycle phase. A different display of the same data is shown using boxplots in Fig. 2C. Boxplots cannot display the bimodal shape of methylation, but allows for better visual comparison of the different samples; the conclusion is unchanged.

We then asked if there was an change specifically localized inside or outside hypomethylation blocks identified in colon cancer^17^(Fig. 2D). As expected we observe lower levels of DNA methylation inside the blocks, but there is no localized change either inside or outside these regions.

**Figure 3:**
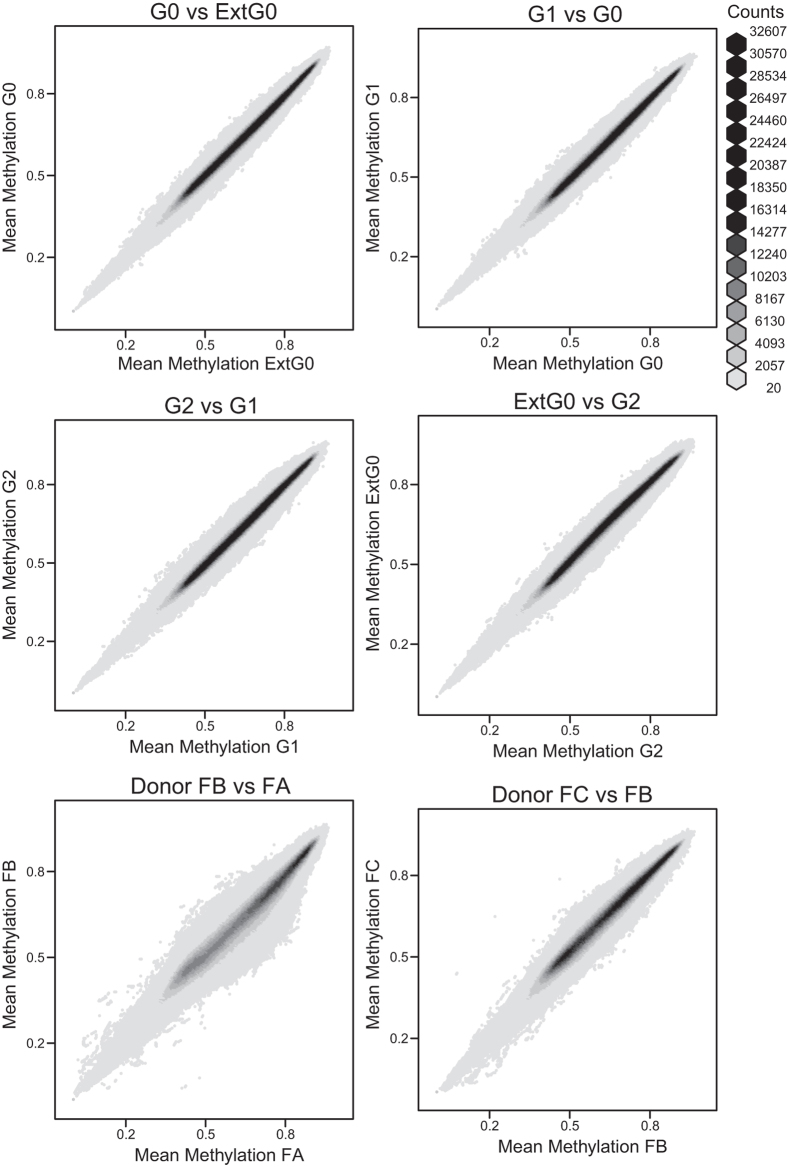
Global methylation is highly correlated between cell cycle phases. Shown is hexagonal binning of mean, high-frequency smoothed methylation values per CpG for all samples in G0 versus all samples in extended G0, all samples in G1 versus all samples in G0, all samples in G2 versus all samples in G1, all samples in extended G0 versus all samples in G2, for all samples from donor FB versus all samples from donor FA, for all samples from donor FC versus all samples from donor FB. The last two comparisons gives a measure of biological variability. For clarity, bins containing less than 20 CpGs are not shown.

We next computed, for each CpG, the average methylation across the three donors, and compared this average methylation between cell cycle phases (Fig. 3). We found a strong correlation between the mean methylation in each cell cycle phase across all comparisons. For comparison we computed the average methylation for each phase of the cell cycle, for each donor. The difference between cell cycle phases is smaller than the difference between donors, indicating that the observed differences are within the margin of biological variability.

The distributions examined above are computed across the entire methylome and smaller, focal changes do not necessarily have an impact on genome-wide measurements. We therefore used BSmooth to identify large and small scale changes associated with cell cycle (Methods), by performing pairwise tests between any two cell cycle phases. We assessed significance of these changes by permutation, which is a conservative approach for this analysis. We found no large scale changes (blocks of either hyper or hypo methylation) using this approach. For each comparison a small number of small DMRs were identified in each comparison but none of these DMRs had a family-wise error rate less than 10% using permutation testing; ie. no DMRs were significant.

## Discussion

In summary, we used low coverage whole genome bisulfite sequencing to demonstrate consistent levels of DNA methylation across phases of the cell cycle in human primary fibroblasts. We used flow sorting to isolate resting and actively dividing cells from low passage primary cell culture and also examined the same cells after one week of arrest. We observed a very strong correlation between methylation in each examined cell cycle phase within each donor’s cells and between donors, with no conclusive evidence of genome wide change associated with either replication or quiescence.

This finding is informative for interpreting large-scale changes in methylation identified in other settings, including colon cancer, EBV immortalization and cellular senescence. We previously observed widespread hypomethylated blocks associated with colon cancer and EBV transformation^17^,^18^,^19^. Intriguingly, these hypomethylated blocks generally involve highly methylated regions shifting to intermediate levels of methylation, indicating that these regions are more variably methylated within the cell population examined. Both of these conditions are associated with increased cell proliferation, and thus an increased percentage of Ki67+ and G2 cells within the population studied, which we hypothesized could explain such heterogeneity. However, our results indicate that these changes alone would not lead to the observed widespread hypomethylation.

Of primary concern for interpretation of this study is whether we have sufficient power to detect changes in DNA methylation. Power is determined by sample size and to a lesser extent sequencing depth. This question was recently examined by Ziller *et al.*, where it is demonstrated that when analyzing 3 replicates, 5–10X coverage detects ~70% of the small DMRs identified in analysis of higher coverage data^27^. Thus the identification of no significant small DMRs in our study does not necessarily indicate that no small DMRs are present, but that the number of DMRs is at least small.

A recent study by Cruikshanks *et al.* implicates proliferative history in the development of hypomethylated blocks^21^. In that study they suggest that mislocalization of DNMT1 in late passage cells contributes to loss of maintenance methylation. Here, we sough to specifically address the affects of cell cycle independent of this potential change and thus used early passage primary cells for our analysis. Our results indicate that proliferation, as measured by Ki67 staining or 4N DNA content, in low passage fibroblasts is not sufficient for this change. It is possible that our findings would be different in late passage cells, as Cruikshanks *et al.* indicate defects in DNMT1 localization only in late passage cells.

We examined cells that were arrested by contact inhibition for one week in order to determine if methylation changes would be introduced or accentuated by an extended exit from the cell cycle, similar to many somatic cell types *in vivo.* We did not observe any large-scale change in methylation associated with the extended cell cycle arrest. However, this length of contact inhibition is much shorter than the length of time many somatic cells spend without dividing, so it remains possible that global methylation changes observed *in vivo* may be linked to extended exit from the cell cycle.

Two previous studies examining global methylation levels across the cell cycle have focused on the dynamics of methylation maintenance during S phase, with an early work reporting hypermethylation during DNA replication and a contradictory recent work showing a linear relationship between DNA content and 5 mC signal during replication^15^,^16^. We did not examine S phase, as using partially replicated DNA would be particularly challenging to accurately measure methylation levels using low coverage sequencing data. While Desjobert *et al.* report a lag in the time at which they detect maximum DNA content and maximum methylation signal in G2/M^16^, our data shows at most a 1% change in mean methylation in G2 cells, indicating that if such a lag exists, effects on the methylome of G2 cells are small.

Interpretation of our data is also limited by the use of a single cell type. We chose to use low passage primary fibroblasts to make our data relevant to the study of primary tissue samples. However these cells are less proliferative than ES or cancer cell lines and our data indicate that they have significant levels of intermediate methylation. It is possible that proliferation or cell cycle dependent changes may be present in other cell types. It is intriguing that we identify significant levels of intermediate methylation even in cultured primary cells sorted based on cell cycle. While our sorting demonstrates that each population is homogenous with regard to size, complexity and DNA content, the intermediate methylation suggests that heterogeneity is present even within these populations, similar to that observed in other studies of primary cells^28^. The factors underlying this heterogeneity remain an interesting area for future investigation.

## Methods

### Tissue Culture

Anonymized primary dermal fibroblasts were purchased from Gibco, ATCC and Lonza (information in Table 1), the authors had no interaction with the donors or identifying information and this is not considered human subjects research as defined by the Code of Federal Regulations. Cells were maintained in Dulbecco’s Modified Eagle Medium DMEM (Life Technologies) supplemented with 15% FBS (Gemini BioProducts) and 1X Pen/Strep (Gibco). Cells were incubated at 37°C with 5% CO_2_.

### Fluorescence-activated Cell Sorting

Passage 4 cells were detached using trypsin (Life Technologies). Trypsin was neutralized using trypsin neutralization buffer (ATCC), washed, fixed in cold 75% ethanol and stored at -20°C. Fixed cells were washed in PBS containing 1% FBS, 0.09% NaN_3_. Cells were stained with FITC conjugated Mouse Anti-Human Ki67 (clone B56) and 25 ug/mL propidium iodide solution (Sigma). Fluorescence activated cell sorting analysis was performed using a Beckman Coulter MoFlo Cell Sorter.

### Whole Genome Bisulfite Sequencing

1% unmethylated Lambda DNA (Promega, cat # D1521) was spiked in to genomic DNA to monitor the bisulfite conversion efficiency. 50–100 ng of genomic DNA was fragmented to a target peak of 300-400 bp using the Covaris S2 Focused-ultrasonicator in a 50 μl volume according to the manufacturer’s instructions.

The fragmented DNA was converted to end-repaired, adenylated DNA using the NEBNext Ultra End Repair/dA-Tailing Module (New England BioLabs, cat # 7442L). Methylated adaptors (NEBNext Multiplex Oligos for Illumina; New England BioLabs, cat # E7535L) were ligated to the product from the preceding step using the NEBNext Ultra Ligation Module (New England BioLabs, cat # 7445L). The resulting product was size-selected as described in the manufacturer’s protocol by employing modified AMPure XP bead ratios of 0.4X and 0.2X in order to select for an insert size of 300–400 bp.

After size-selection the samples were bisulfite converted and purified using the EZ DNA Methylation- Gold Kit (Zymo Research, cat # D5005). Bisulfite converted libraries were PCR amplified and indexed using primers from the NEBNext Multiplex Oligos for Illumina module (New England BioLabs, cat # E7535L) and the Kapa HiFi Uracil+ PCR system (Kapa Biosystems, cat # KK2801). PCR enrichment was performed with the following cycling parameters: 98°C for 45 sec followed by 10 cycles at 98°C for 15 sec, 65°C for 30 sec, 72°C for 30 sec and a final extension at 72°C for 1 min. The PCR enriched product was cleaned up using 1X AMPure XP beads (Beckman Coulter, cat # A63881).

The resulting libraries were sequenced at a 2 × 100 bp read length on the Illumina HiSeq2000 platform using v3 chemistry according to the manufacturer’s protocol.

### Data Analysis

All analyses were performed using Bioconductor and R 3.2^29^,^30^. To process sequencing data, we ran the BSmooth^26^ bisulfite alignment pipeline on the 100-by-100 bp HiSeq 2000 paired end sequencing reads obtained for each sample, using Bowtie2 version 2.1.0^31^ and the hg19 build on the human genome as well as the genome for lambda phage. Table 2 summarizes the alignment results. After alignment, BSmooth was used to extract read-level measurements, summarized in Table 2. We filtered out measurements with mapping quality <20 or nucleotide base quality <10. We used M-bias plots^26^ for quality control of our samples. Based on the M-bias plots we removed 10 nucleotides from the 5’ end of each rate (both mates) when extracting read-level measurements.

BSmooth was used to identify large hypomethylated blocks as described in detail previously^17^,^18^,^26^. CpGs with coverage of 2 or greater in 2 out of the 3 samples in each sample group were included in the analysis. We used the same cutoffs used in studies of cancer and EBV transformation, specifically a t-statistic cutoff of (-2, 2) for block finding and (-4.6, 4.6) for DMR finding. Candidate blocks were filtered by size, using a minimum cutoff of 100,000 bp. No candidates passed the width cutoff for block finding. Candidate DMRs were filtered by CpG number and magnitudes, using a minimum cutoff of 3 CpGs and >10% difference. A FWER statistic for candidate DMRs was calculated by performing 10 permutations of sample group assignments, as previously described^18^. The p-values are constrained to have values of x/10, with x an integer, but have already been corrected for searching genome-wide. No small DMRs were considered significant using this procedure.

## Additional Information

**Accession Codes**: Sequencing data is available on GEO under accession GSE68657.

**How to cite this article**: Vandiver, A. R. *et al.* DNA methylation is stable during replication and cell cycle arrest. *Sci. Rep.* **5**, 17911; doi: 10.1038/srep17911 (2015).

## Figures and Tables

**Table 1:** Description of primary samples.

Donor	Supplier	Catalog #	Lot	Donor Age	Donor Gender	Donor Race	Days in Culture
FA	Gibco	C-013-5C	1474560	36	Female	Caucasian	18
FB	Lonza	CC-2511	352805	40	Female	Caucasian	21
FC	ATCC	PCS-201-012	61447289	34	Female	Caucasian	23

**Table 2:** Summary of whole genome bisulfite sequencing alignment.

Sample	# paired-ends reads sequenced	# ends sequenced (reads x2)	# ends aligned	Alignment rate	Covered_ CpGs	Coverage	Mean Depth	Conversion Rate
FA_Ext_G0	183161558	366323116	299150337	0.82	25514228	0.90	7.21	0.997
FA_G0	227927743	455855486	372489752	0.82	25647479	0.91	8.60	0.997
FA_G1	145296086	290592172	239512225	0.82	24826013	0.88	5.28	0.9971
FA_G2	169775086	339550172	276277216	0.81	25322198	0.90	6.60	0.9971
FB_Ext_G0	165662207	331324414	269406543	0.81	25350618	0.90	6.29	0.9971
FB_G0	173184150	346368300	280062174	0.81	25393776	0.90	6.92	0.997
FB_G1	137373738	274747476	224625545	0.82	25312601	0.90	5.69	0.9968
FB_G2	139872847	279745694	224734195	0.80	25313403	0.90	5.61	0.9971
FC_Ext_G0	186215053	372430106	302409275	0.81	25647389	0.91	7.54	0.997
FC_G0	183961564	367923128	299798716	0.81	25665135	0.91	7.48	0.997
FC_G1	180202865	360405730	294426472	0.82	25602198	0.91	7.13	0.9969
FC_G2	159859928	319719856	256338433	0.80	25511322	0.90	6.51	0.9968
